# Supplementary material for: Vertex model with internal dissipation enables sustained flows
Source: Nat Commun. 2025 Jan 9;16:530. doi: 10.1038/s41467-025-55820-2 (PMC11718050; doi:10.1038/s41467-025-55820-2)
Supplement: Supplementary file 1 — Supplementary Information [file 41467_2025_55820_MOESM1_ESM.pdf]

Supplementary Information

Vertex model with internal dissipation enables sustained flows

Jan Rozman, Chaithanya K. V. S., Julia M. Yeomans, and Rastko Sknepnek

Supplementary Figures

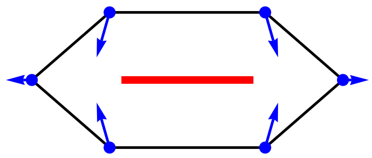

Supplementary Figure 1. Schematic of the active forces. Blue arrows show active forces on the vertices of an elongated cell arising from the cell's stress tensor. Red line shows the director.

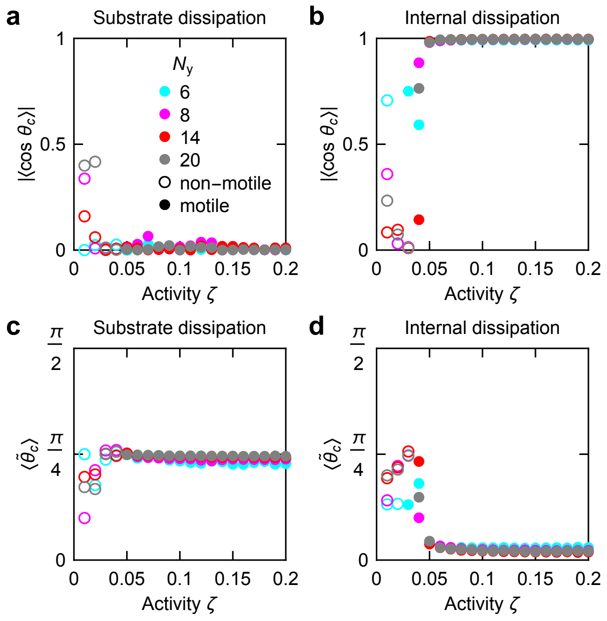

Supplementary Figure 2. Comparison of models with substrate and internal dissipation. **a&b**) Absolute value of the average cosine of the angle θ_c between cell velocity and the x axis for the model with substrate friction (**a**) and the model with internal dissipation (**b**). **c-d**) Average of angle $\tilde{\theta}_c$ (θ_c confined to the range $[0, \pi/2]$) for the model with substrate friction (**c**) and the model with internal dissipation (**d**). Legend in panel **a** applies to the entire figure. Empty circles on all panels correspond to simulations where final MSD < 1. See Methods for details of how all values were determined.

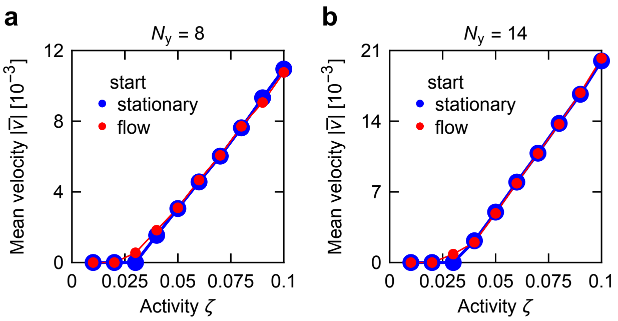

Supplementary Figure 3. Comparison between channels starting from a stationary and a flow configuration. Mean velocity along x as a function of activity for two different possible starts, either starting with the final activity and seeing if a flow develops (stationary start) or first running the simulation at $\zeta = 0.1$ so that a flow configuration develops (see Methods) and then reducing the activity to a final value (flow start); using $N_y = 8$ (**a**) and $N_y = 14$ (**b**).

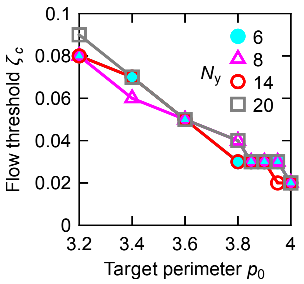

Supplementary Figure 4. Threshold activities decrease with increasing target perimeter. Threshold activity for unidirectional flows (Methods) for different target perimeters p_0 and channel widths.

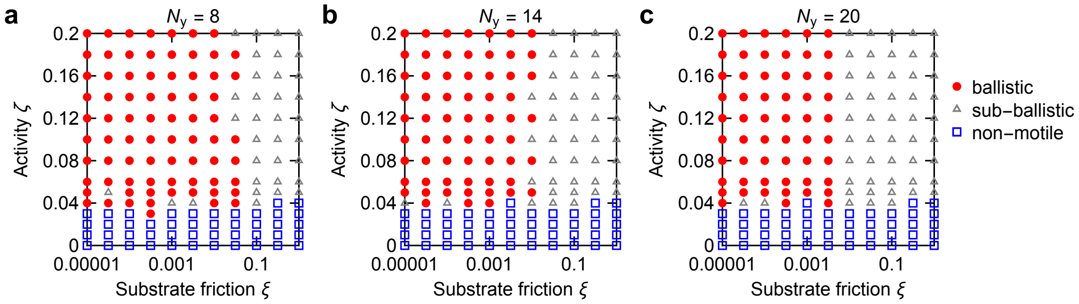

Supplementary Figure 5. Phase diagrams of the model. **a-c**) Phase diagram spanning substrate friction and activity using $N_y = 8$ (**a**), $N_y = 14$ (**b**; same as main text), and $N_y = 20$ (**c**); $\eta = 1$ on all panels.

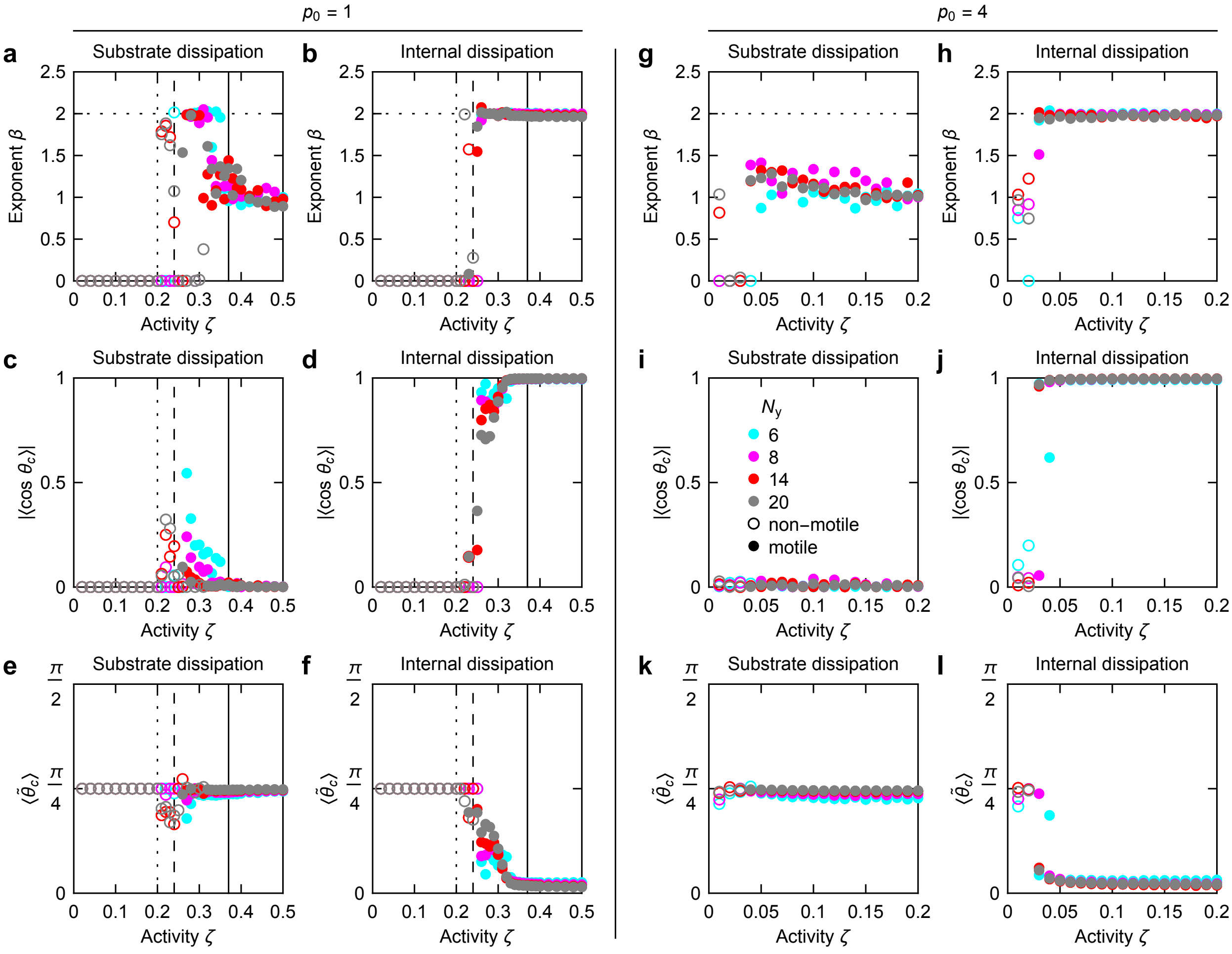

Supplementary Figure 6. Comparing substrate and internal dissipation models at different target perimeters. **a-f**) For $p_0 = 1$ (i.e., far in the solid phase of the passive model): **a**&**b**) Exponent of at^β fit to MSD for the substrate (**a**) and internal (**b**) dissipation model. **c**&**d**) The absolute value of the average cosine of the angle θ_c between cell velocity and the x axis for the substrate (**c**) and internal (**d**) dissipation model. **e**&**f**) Average of angle $\tilde{\theta}_c$ (θ_c confined to the range $[0, \pi/2]$) for the substrate (**e**) and internal (**f**) dissipation model. **g-l**). For $p_0 = 4$ (i.e., far in the solid phase of the passive model): **g**&**h**) Exponent of at^β fit to MSD for the substrate (**g**) and internal (**h**) dissipation model. **i**&**j**) Absolute value of the average cosine of the angle θ_c between cell velocity and the x axis for the substrate (**i**) and internal (**j**) dissipation model. **k**&**l**) average of angle $\tilde{\theta}_c$ for the substrate (**k**) and internal (**l**) dissipation model. Empty circles on all panels correspond to simulations where final MSD < 1. See Methods for details of how all values were determined. Legend in panel **j** applies to the entire figure. Horizontal dotted line on panels **a**,**b**,**g**,**h** at $\beta = 2$ mark ballistic motion. Vertical dotted, dashed, and full lines on panels **a-f** show $\zeta = 0.2$, 0.24, and 0.37, the reported thresholds for the anisotropic solid, rhombile, and fluid phases of the model, respectively [1].

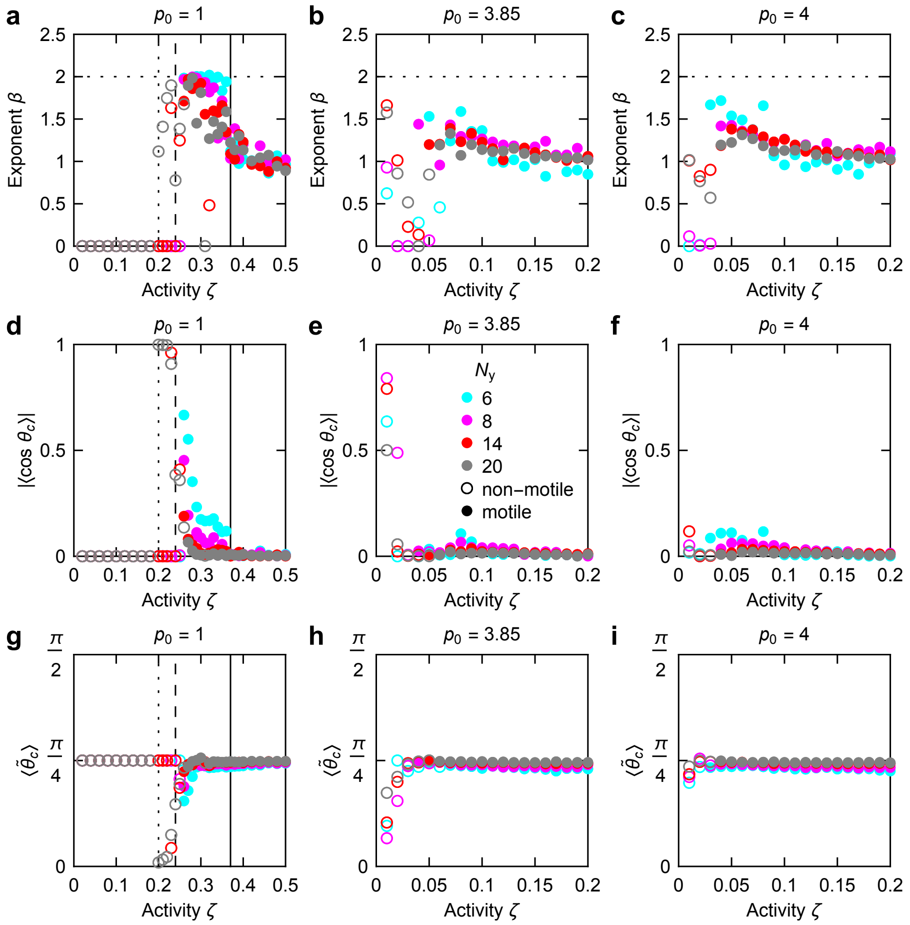

Supplementary Figure 7. Substrate dissipation model starting from a flow configuration. Top to bottom: Exponent of at^{β} fit to MSD for the substrate dissipation model, absolute value of the average cosine of the angle θ_c between cell velocity and the x axis, and average of angle $\tilde{\theta}_c$ (θ_c confined to the range $[0, \pi/2]$) for simulations starting from a flow configuration (Methods) for $p_0 = 1$ (**a**,**d**,**g**), $p_0 = 3.85$ (**b**,**e**,**h**), and $p_0 = 4$ (**c**,**f**,**i**). See Methods for details of how all values were determined. Legend in panel **e** applies to the entire figure. Horizontal dotted line on panels **a-c** at $\beta = 2$ mark ballistic motion. Vertical dotted, dashed, and full lines on panels **a**,**d**,**g** show $\zeta = 0.2$, 0.24, and 0.37, the reported thresholds for the anisotropic solid, rhombile, and fluid phases of the model, respectively [1].

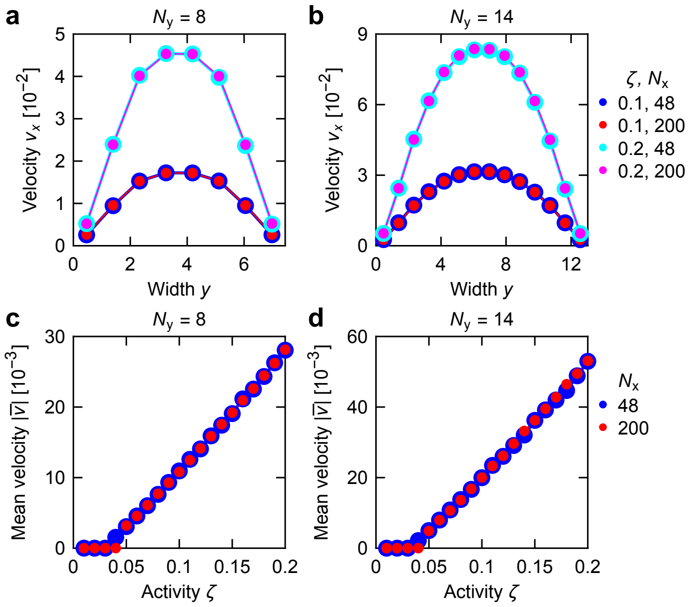

Supplementary Figure 8. Flow profiles at different channel length. a&b) Velocity profile across the channel for shorter ($N_x = 48$) and longer ($N_x = 200$) channels with $N_y = 8$ (**a**) and $N_y = 14$ (**b**) at two different activities. **c&d**) Mean velocity along x as a function of activity for shorter ($N_x = 48$) and longer ($N_x = 200$) channels with $N_y = 8$ (**c**) and $N_y = 14$ (**d**).

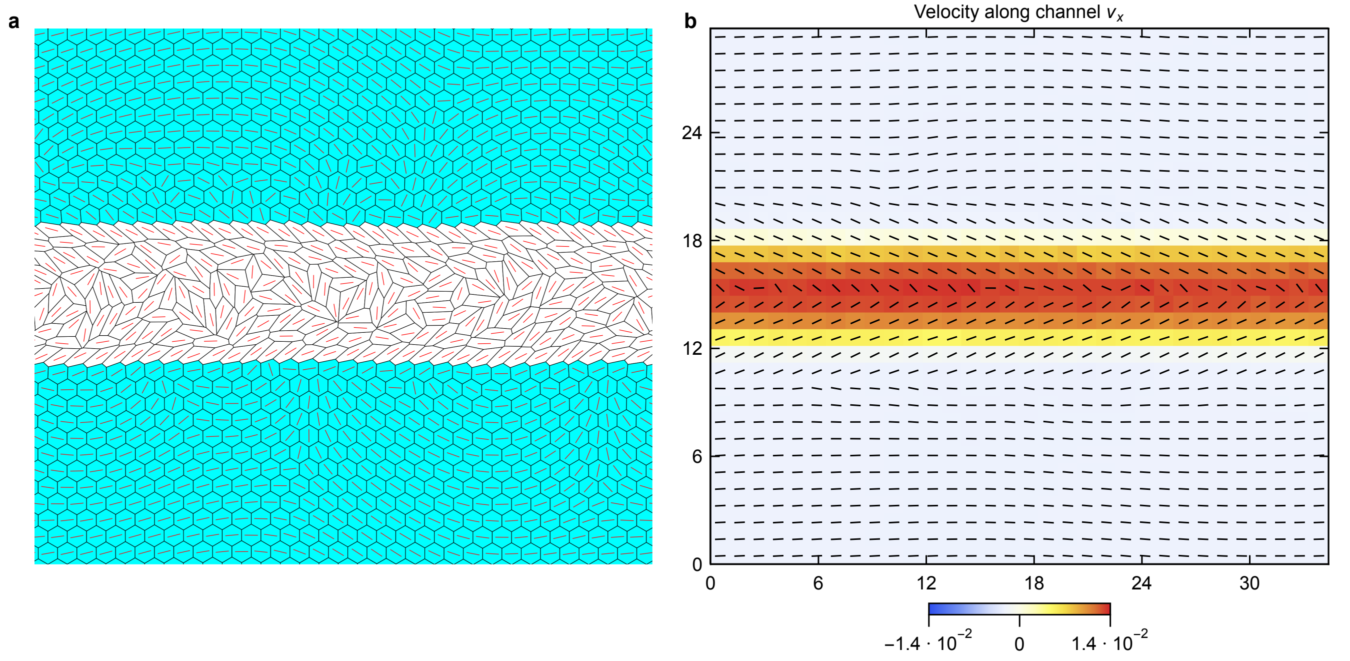

Supplementary Figure 9. Channel flows in a tissue. a&b) Model tissue with periodic boundary conditions shown at $t = 2 \cdot 10^5$ (**a**) with the corresponding velocity and director profiles averaged between $t = 1 \cdot 10^5$ and $t = 2 \cdot 10^5$ in increments of $\Delta t = 500$ (**b**). Softer, active white cells have $p_0 = 3.85$ and $\zeta = 0.1$. Solid, passive cyan cells have $p_0 = 1$ (i.e., far in the solid regime of the passive model) and $\zeta = 0$. Red lines show cell directors in **a**, black lines show averaged cell directors in **b**.

Supplementary References

[1] Lin, S.-Z., Merkel, M. & Rupprecht, J.-F. Structure and rheology in vertex models under cell-shape-dependent active stresses. *Phys. Rev. Lett.* **130**, 058202 (2023).
