# Supplementary material for: Vertex model with internal dissipation enables sustained flows
Source: Nat Commun. 2025 Jan 9;16:530. doi: 10.1038/s41467-025-55820-2 (PMC11718050; doi:10.1038/s41467-025-55820-2)
Supplement: Supplementary file 2 — Description of Additional Supplementary Files [file 41467_2025_55820_MOESM2_ESM.pdf]

Description of Additional Supplementary Files

Supplementary Movie 1

Evolution of a model tissue in a channel using substrate dissipation dynamics with $p_0 = 3.85$ and $\zeta = 0.1$. Simulation runs until $t = 2 \times 10^5$. A subset of cells is coloured to enable tracking.

Supplementary Movie 2

Evolution of a model tissue in a channel using internal dissipation dynamics with $p_0 = 3.85$ and $\zeta = 0.1$. Simulation runs until $t = 2 \times 10^5$. A subset of cells is coloured to enable tracking.

Supplementary Movie 3

Evolution of a model tissue in a channel using internal dissipation dynamics with $p_0 = 3.85$ and $\zeta = 0.04$. Simulation runs until $t = 2 \times 10^5$. A subset of cells is coloured to enable tracking.

Supplementary Movie 4

Evolution of a periodic model tissue in a channel using internal dissipation dynamics. White cells have $p_0 = 3.85$ and $\zeta = 0.1$. Cyan cells have $p_0 = 1$ and $\zeta = 0$. Simulation runs until $t = 2 \times 10^5$. A subset of cells is coloured to enable tracking.
